# Earable TEMPO: A Novel, Hands-Free Input Device that Uses the Movement of the Tongue Measured with a Wearable Ear Sensor

**DOI:** 10.3390/s18030733

**Published:** 2018-03-01

**Authors:** Kazuhiro Taniguchi, Hisashi Kondo, Mami Kurosawa, Atsushi Nishikawa

**Affiliations:** 1 Graduate School of Information Sciences, Hiroshima City University, 3-4-1 Ozukahigashi, Asaminami-ku, Hiroshima 731-3194, Japan; 2 eRCC Co., Ltd., 21-3 Motomachi, Nakaku-ku, Hiroshima 730-8504, Japan; wmlabo@rcc.co.jp; 3 Interdisciplinary Graduate School of Science and Technology, Shinshu University, 3-15-1 Tokida, Ueda, Nagano 386-8567, Japan; 17st104f@shinshu-u.ac.jp; 4 Faculty of Textile Science and Technology, Shinshu University, 3-15-1 Tokida, Ueda, Nagano 386-8567, Japan; nishikawa@shinshu-u.ac.jp; 5 Division of Biological and Medical Fibers, Institute for Fiber Engineering (IFES), Interdisciplinary Cluster for Cutting Edge Research (ICCER), Shinshu University, 3-15-1 Tokida, Ueda, Nagano 386-8567, Japan

**Keywords:** ear canal, tongue, non-invasive, optical measurement, hand-free controller

## Abstract

In this study, an earphone-type interface named “earable TEMPO” was developed for hands-free operation, wherein the user can control the device by simply pushing the tongue against the roof of the mouth for about one second. This interface can be used to start and stop the music from a portable audio player. The earable TEMPO uses an earphone-type sensor equipped with a light emitting diode (LED) and a phototransistor to optically measure shape variations that occur in the external auditory meatus when the tongue is pressed against the roof of the mouth. To evaluate the operation of the earable TEMPO, experiments were performed on five subjects (men and women aged 22–58) while resting, chewing gum (representing mastication), and walking. The average accuracy was 100% while resting and chewing and 99% while walking. The precision was 100% under all conditions. The average recall value of the five subjects was 92%, 90%, and 48% while resting, masticating, and walking, respectively. All subjects were reliably able to perform the action of pressing the tongue against the roof of the mouth. The measured shape variations in the ear canal were highly reproducible, indicating that this method is suitable for various applications such as controlling a portable audio player.

## 1. Introduction

Portable audio players (PAPs) were first sold in 1979 and are still available in 2017 all over the world. With PAPs, users can enjoy music with ease on a daily basis, anytime and anywhere. The performance of PAPs has been improved, and new functions have been added, such as smaller size, change in storage mediums, additional communication functions, improved tone quality, additional display devices, and improved operability. However, much room still exists for improving the operability of PAPs. This is because PAPs must currently be manually manipulated via the user interface on the main unit or through the use of a remote controller mounted on the earphone cable. Thus, users who do not have full use of their hands, such as those holding baggage or grasping a strap while standing on a train or bus or people with physical disabilities, have difficulty operating PAPs. Therefore, we believe that adding a hands-free operation function to PAPs is necessary to improve their operability. 

Our group has previously investigated hands-free operation components in wearable devices [1,2,3,4,5], including device operation via the user’s blinking or back-teeth chewing; however, for the hands-free operation of PAPs, the movement of the tongue would be better suited than blinking or back-teeth chewing, because if a PAP is used while walking, the action of closing the eyes can be dangerous, since sight is momentarily lost and the action of back-teeth chewing is dangerous, as the user may mistakenly bite his or her own tongue. In addition, even users who have impaired vision, damaged teeth, or reduced jaw strength or those with physical disabilities in their arms or legs can perform tongue movements. Moreover, since the operation does not depend on language, it can be used worldwide.

One method of estimating the movement of the tongue for hands-free interfaces involves invasively attaching a permanent magnet to the tip of the tongue and tracking the movement of the magnet using a magnetic sensor attached to the cheek or another nearby position [6,7,8]. However, noninvasive methods are more desirable for hands-free operation of PAPs. A noninvasive method for estimating the movement of the tongue involves attaching the electrodes to the cheek or jaw and using electromyography (EMG) to measure the electrical signals generated by the tongue movements [9,10]. However, the electrodes must be applied to the cheeks or jaws, which is inconvenient and unaesthetic; thus, this method is not suitable for easy, everyday, hands-free operation of PAPs. Other studies have used a mouthpiece-type EMG sensor to measure the EMG signal within the oral cavity; these measurements can be used to estimate the movement of the tongue and can be applied to the computer interface [11]. Dental retainers with built-in light sensors [12] and joysticks for the tongue [13] have also been used instead of mouthpieces to measure tongue movements for people with quadriplegia; one study using a joystick designed for persons without any disability targeted actors wearing costumes [14]. However, for PAP operation, not placing a sensor in the mouth is desirable. 

Regarding studies of hands-free PAP operation, one suitable method that has been previously proposed is measuring pressure changes in the outer ear caused by the movement of the tongue by using a microphone built in the earphone [15,16,17]. Using this approach, four types of tongue movements could be distinguished from the obtained measurements. The technique used in a previous study [16] helped attain an average accuracy of 97% among eight subjects. However, when applying this method to the operation of a PAP, evaluating erroneous operations other than ones caused during resting, such as the movement of the tongue while chewing and erroneous operations due to the associated shape variations in the external auditory meatus while walking, is also necessary. These evaluations, however, have not been conducted.

In this study, our previously developed method for hands-free operation of a wearable device is applied to the operation of a PAP. Herein, we discuss the concept and working of the earphone-type interface named “earable TEMPO” that can be controlled via the movement of the tongue to start and stop the media on a PAP. In the evaluation experiment, the performance was evaluated while resting, gum chewing (mastication), and walking.

## 2. Materials and Methods

### 2.1. Concept

PAP users often listen to music while engaging in other activities such as sitting in a chair while studying, walking, or dining. Thus, the user interface for hands-free operation of a PAP should be easy to operate so that it can be used while engaging in other activities. In addition, the installation of the interface can be made easier by integrating it with earphones.

**Figure 1:**
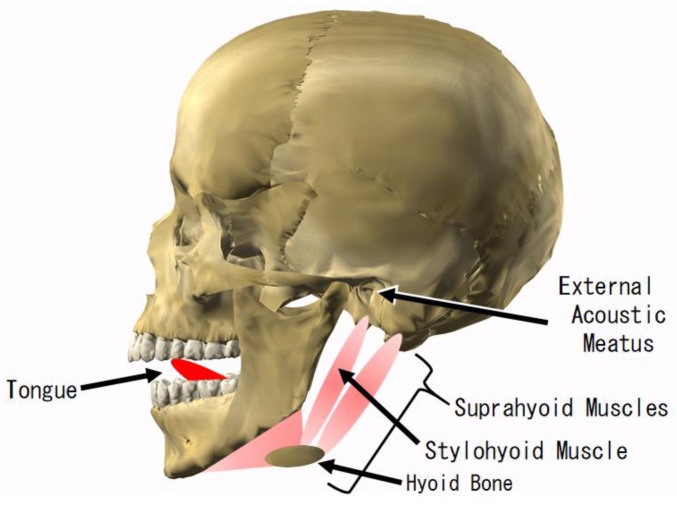
Anatomy of the tongue and external acoustic meatus. When the user pushes the tongue against the roof of the mouth, the suprahyoid muscles, including the stylohyoid muscle, expand and contract. The shape of the adjacent external acoustic meatus changes as a result of this expansion and contraction.

Therefore, our research and development is on an earphone-type interface, the earable TEMPO, that can perform one-bit switch operation of the PAP hands-free to toggle the music on and off simply by pushing the tongue against the roof of the mouth for about one second. When the user pushes the tongue against the roof of the mouth for about one second, the suprahyoid muscles, including the stylohyoid muscle, expand and contract. The shape of the adjacent external acoustic meatus changes as a result of this expansion and contraction (Figure 1). Thus, the movement of the tongue can be optically and noninvasively measured using an earphone-type sensor. Hereafter, the action of pressing the tongue against the roof of the mouth for about one second is referred to as TEMPO (Tongue and Ear Movement for PAP Operation).

TEMPO is a movement that can be easily performed without special training. Furthermore, since the operation is not one that is normally performed in daily life, one rarely makes incorrect device operations. In addition, a person is less likely to accidentally bite his or her tongue during TEMPO. Finally, TEMPO can be performed while walking or during meals and is not noticeable to other people.

While performing TEMPO, the user can simply imagine that “a push button switch is attached to his or her maxilla to play and stop music” and can move the tongue as if he or she is going to press or release the button switch with the tongue. Such imagination helps the user understand the operating method.

The earable TEMPO measures the shape variation of the external acoustic meatus via the earphone-type sensor that is attached to the ear. It calculates the degree of similarity between the measured signal and predetermined data that matches the patterns of TEMPO, categorizing whether the variation is TEMPO. When it is determined that the user has performed TEMPO, the PAP operation is performed. The details will be described below.

### 2.2. Hardware Design

**Figure 2:**
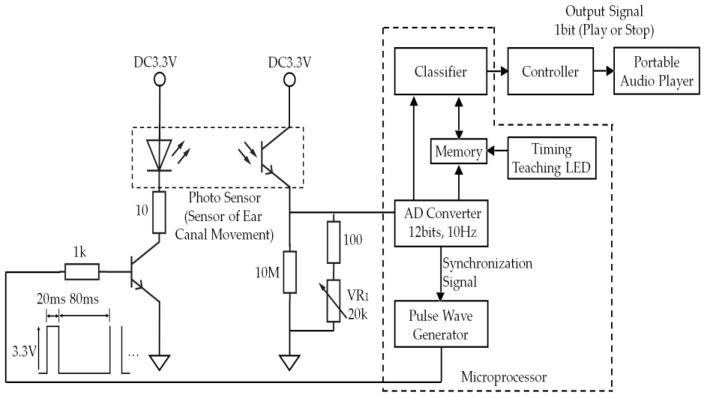
Construction of the electronic circuit of the earable TEMPO and the appearance of the earphone-type sensor. The earable TEMPO optically measures the shape variation of the auditory canal via an earphone-type sensor inserted into one ear. The switch manipulates the PAP through the controller when a classifier judges that the user has performed TEMPO. The offset voltage of the output of the earphone-type sensor can be adjusted with the variable resistor VR_1_. A pulse wave generator is attached to the LED of the earphone-type sensor to control the light emission of the LED. The microprocessor comprises five components: an AD converter, a pulse wave generator, a timing-teaching LED, memory, and a classifier.

**Figure 3:**
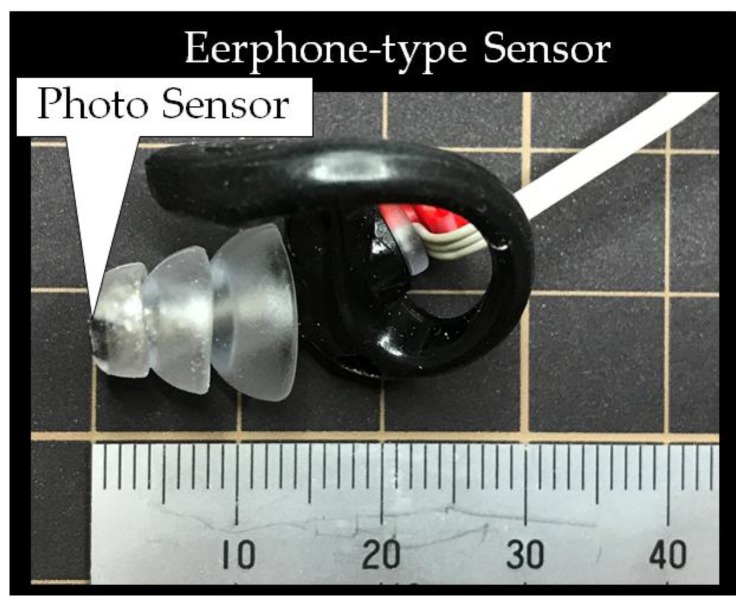
Earphone-type sensor for the right ear. The earphone-type sensor is equipped with a small optical sensor comprising a QRE 1113 sensor (Fairchild Semiconductor International Inc., San Jose, CA, USA). An infrared LED and phototransistor is equipped in the optical sensor. The size of the area that is used for the earphone-type sensor to be inserted into the ear canal was based on a medium sized commercially available earphone. Speakers were not mounted onto the sensor in this study, because the objective here is only to evaluate the operating performance of a PAP using a prototyped earphone-type sensor; however, speakers can be added in future applications.

**Figure 4:**
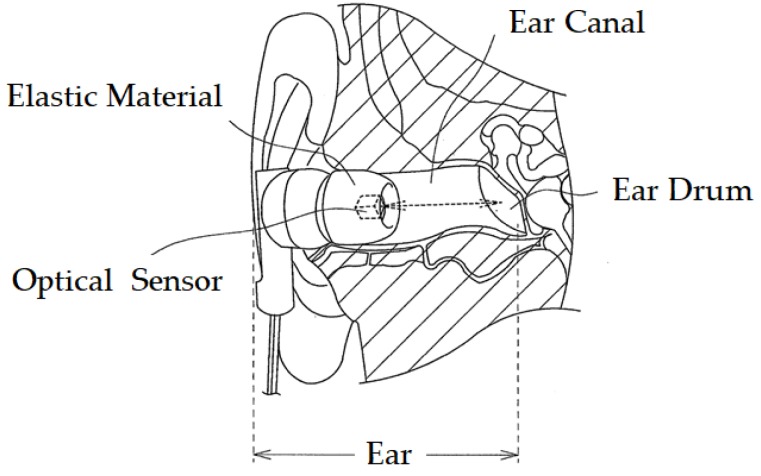
Measurement principle for changes in the shape of the ear canal. The earphone-type sensor receives the light emitted from the optical distance sensor that is reflected back by the eardrum and ear canal. During movement of the tongue, the shape of the ear canal changes, which alters the distance between the optical distance sensor and the eardrum and ear canal. The amount of light received changes over time in association with this change in distance.

The system configuration of the earable TEMPO is shown in Figure 2. It is a device where an earphone-type sensor for the right ear (Figure 3) is used to measure the movement of the user’s external auditory meatus and perform a one-bit switch operation (starting and stopping) of a PAP based on the measurement results. The picture right below Figure 1 shows the appearance of the earphone-type sensor of the earable TEMPO. The earphone-type sensor is equipped with a small optical sensor comprising a QRE 1113 sensor (Fairchild Semiconductor International Inc., San Jose, CA, USA). An infrared LED and phototransistor is equipped in the optical sensor. By transmitting infrared light into the external acoustic meatus by LED and receiving the reflected light by the phototransistor, the movement of the external acoustic meatus is measured (Figure 4). While conventional methods of measuring the movement of the ear canal include the use of a microphone [15,16,17], the use of an optical sensor has an advantage where there is less influence from environmental sounds, and it is not necessary to tightly seal the ear hole. The output of the optical sensor increases when the amount of light reflected from the object increases and decreases as the amount of reflected light decreases. The offset voltage of the sensor output can be adjusted using the variable resistor VR_1_. A pulse wave generator is attached to the LED to control the optical emission of the LED and is synchronized with the analog-digital (AD) converter connected to the earphone-type sensor. By making the structure to emit light only during the AD conversion period, the light intensity of the LED can be enhanced, which is expected to lead to a higher signal-to-noise (SN) ratio. The reason for this is that the impact of infrared rays, contained in ambient light, that pass through the skin in the vicinity of the outer ear can be minimized. The size of the area that is used for the earphone-type sensor to be inserted into the ear canal was based on a medium sized commercially available earphone. Speakers were not mounted onto the sensor in this study, because the objective here is only to evaluate the operating performance of a PAP using a prototyped earphone-type sensor; however, speakers can be added in future applications.

The analog signal measured with the earphone-type sensor is converted into digital signals using an AD converter with a resolution of 12 bits at a sampling frequency of 10 Hz. The converted signal corresponding to the last two seconds is stored in the memory by the FIFO method. The data recorded in the memory is a set of 20 values, *v_i_*(*i* = 0, 1, 2, ..., 19), representing the voltages measured by the earphone-type sensor at intervals of 0.1 s.

A classifier is used to distinguish between the TEMPO and other movements. When TEMPO is recognized, a controller starts or stops playing music. The algorithm used in the classifier is described in the next section (Section 2.3).

The timing-teaching single LED is used to teach the appropriate timing of TEMPO to the user and comprises one LED, as discussed in the evaluation experiments under Section 3.

In this study, a microprocessor, mbed LPC 1768 (Switch Science Inc., Tokyo, Japan), was used and was operated with custom software (C language). The microprocessor comprises five components: an AD converter, a pulse wave generator, a timing-teaching LED, memory, and a classifier. Although not shown in Figure 1, a surfacePro 3 tablet terminal (Microsoft Corp., Redmond, WA, USA) was connected to the mbed LPC 1768 via USB, and the data from the memory device was simultaneously stored in the tablet. An iPhone 7 (Apple Inc., Cupertino, CA, USA) was used as the PAP. The PAP, by modifying the resistance value of the earphone jack, can switch between playing and stopping. The controller changes the resistance value on the PAP earphone jack, according to the 2-bit signal obtained from the Classifier. The connection between the controller and PAP uses Apple Lightning (Apple Inc., Cupertino, CA, USA).

### 2.3. Algorithm

The classification algorithm determines whether the user has performed TEMPO or other operations and sends the result to the PAP controller as a one-bit signal (1 when the action performed by the user is classified as TEMPO and 0 otherwise). When the PAP is in the stop state, if 1 is sent from the classifier, the PAP controller outputs a signal to start the music; conversely, if the music is playing when a signal of 1 is received, the music stops. Thus, the user can recognize whether the operation was effective or not based on the music from the PAP.

To conduct classification, the algorithm calculates the correlation coefficient between the ground truth stored in the memory and the value measured by the earphone-type sensor at each time point (every 0.1 s). An action at a single time point is classified as TEMPO if the correlation coefficient is 0.9 or more. Ground truth *g_i_*(*i* = 0, 1, 2, ..., 19) is given by Equation (1).

(1) $$g_{i}=Me(e_{i}^{1},e_{i}^{2},e_{i}^{3},\ldots ,e_{i}^{10}),\;i=0,\;1,\;2,\;\ldots ,\;19$$

Here, *Me* is a function that seeks the median value of 10 data items *e_i_*^1^, *e_i_*^2^, ...., *e_i_*^10^, *e_i_^j^*(*i* = 0, 1, 2,..., 19) that is one time of the TEMPO measurement data set *v_i_^j^*(*i* = 0, 1, 2,..., 19) normalized with Equation (2). TEMPO is carried out 10 times on the subjects, and the data each time is expressed with the suffix *j* = 1, 2, 3,..., 10.

(2) $$e_{i}^{j}=\frac{v_{i}^{j}-min_{j}}{max_{j}-min_{j}},\;i=0,\;1,\;2\;,\ldots ,\;19,\;j=1,\;2,\;3,\;\ldots ,\;10$$

in which *max_j_* and *min_j_* are the maximum and minimum of the set of values denoted by *v_i_^j^*(*i* = 0, 1, 2,..., 19) that were measured using the earphone-type sensor.

## 3. Evaluation Experiments

### 3.1. Subjects

The subjects were five healthy volunteers (men and women aged 22–58 with an average age of 33.6) who wear medium-sized earbuds, do not suffer from ear pain or fatigue, and are not undergoing medical treatment. The subjects are referred to as subjects A, B, C, D, and E. The study protocol was approved by the Ethics Committee of Shinshu University’s Human Science Research. All subjects gave informed consent prior to participating in the study. In all experiments, the earphone-type sensors were cleaned and disinfected with ethanol before and after use.

### 3.2. Measurement of Data for Learning to Create TEMPO Ground Truth

An earphone-type sensor was attached to the right ear of the subject, and the subject was asked to perform TEMPO each time the timing-teaching LED was lit. Measurements were recorded using the earphone-type sensor for ten trials, each comprising the LED being on for one second and off for two seconds. Ground truth were extracted from this data for each subject using the method described in Section 2.3.

### 3.3. PAP Operation Experiment

The subject was asked to repeat the TEMPO ten times in accordance with the lighting of the timing-teaching LED. In addition, measurements were taken while resting for three minutes, masticating 100 times, and walking for 80 s (the total time needed to repeat the TEMPO ten times each for about two seconds with a total idle period of 60 s between TEMPOs). The timing for the timing-teaching LED was varied for each type of experiment. In the mastication experiment, the subject was asked to chew gum before and after the TEMPO period but not during TEMPO.

In this study, the appropriate ground truth for each subject was stored in the memory of the experimental setup in advance. Then, for each measurement obtained by the earphone-type sensor, the correlation coefficient with the ground truth was calculated; if the value was 0.9 or greater, the music from the PAP was started or stopped. Put another way, a continuously sliding window and ground truth signal are compared, and if the two are similar, the song is played/stopped with PAP.

The lighting state of the timing-teaching LED, the measured data from the earphone-type sensor, and the correlation coefficient between the measured data and the ground truth were automatically recorded in the tablet terminal connected to the system as shown in Figure 1.

## 4. Results

**Figure 5:**
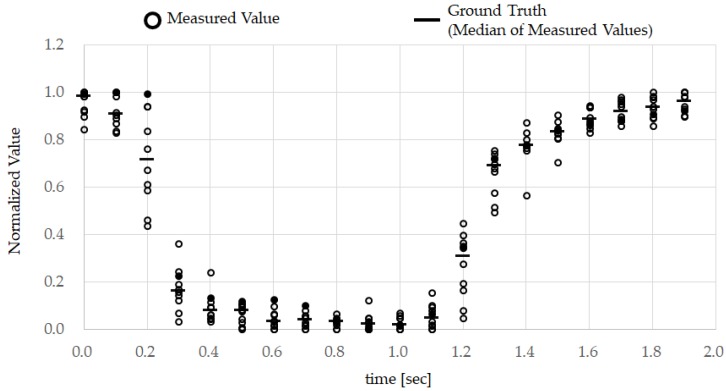
Ground truth obtained from subject A: the subject repeated TEMPO ten times, while the movement of the external acoustic meatus was measured by the earphone-type sensor. The obtained data was normalized, and the median values from the normalized data from each time were extracted and used as the ground truth as described in Section 3.2.

**Figure 6:**
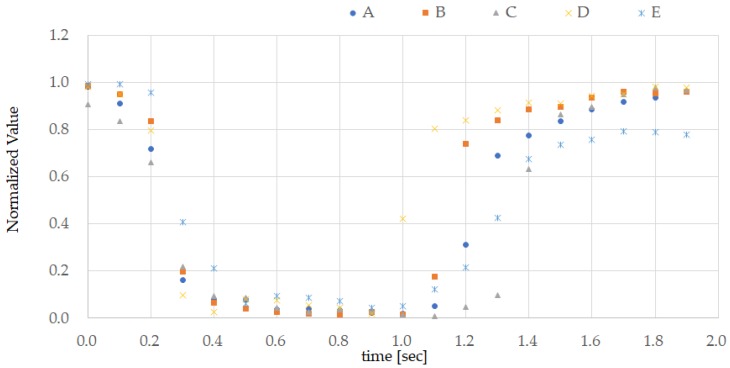
Ground truth of the persons (A–E) being tested. Ground truth is also obtained for the persons (B–E) being tested in the same manner as in Figure 5, and the results are superimposed and displayed. The measured values were obtained from the experiment discussed in Section 3.2.

**Figure 7:**
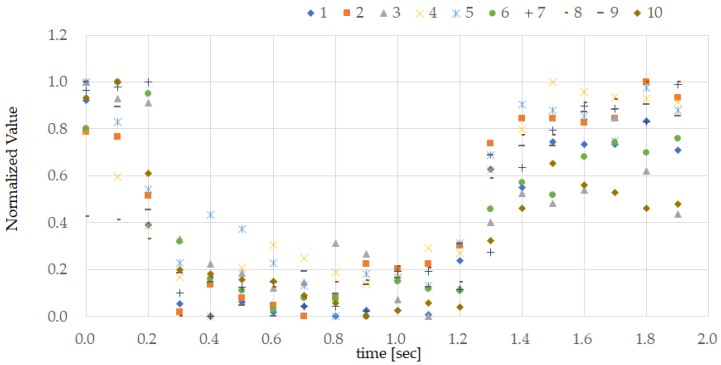
Measured values of person A while walking. The result is obtained by normalizing the measurement values of the earphone-type sensor when person A performs the TEMPO 10 times while walking. These measurement values were obtained from the experiment discussed in Section 3.3.

Figure 5 shows the ground truth obtained from subject A and the set of data obtained by the earphone-type sensor that was used for pattern matching, comprising 20 time points taken at 0.1 s intervals. The ground truth was then created by calculating the median values as defined in Equation (1) from the ten normalized datasets as defined in Equation (2). While some approaches are based on the mean value, the average value is more easily influenced by outliers, while the median value is more resistant to noise. Figure 6 shows the ground truth for all test subjects. Figure 7 shows the measurements from the earphone-type sensor for the ten TEMPO repeats conducted by subject A while walking.

The results of the PAP operation experiment are shown in Table 1. The accuracy of these trials was calculated according to Equation (3), in which an accuracy value close to 1 indicates that the TEMPO classification was correct.

accuracy = (TP + TN)/(TP + FP + FN + TN),(3)

Here, TP (true positive) represents the number of times that the classifier correctly classifies a movement as TEMPO (i.e., the level of similarity was 0.9 or more), FP (false positive) represents the number of times that the classifier wrongly classifies a movement as TEMPO, TN (true negative) represents the number of times that the classifier correctly does not classify non-TEMPO movement as TEMPO (i.e., the level of similarity was less than 0.9), and FN (false negative) represents the number of times that the classifier fails to classify TEMPO as TEMPO. The precision and recall shown in Table 1 are calculated using Equations (4) and (5), respectively.

precision = TP/(TP + FP),(4)

recall = TP/(TP + FN),(5)

Here, precision is taken as the ratio of the number of times that the classifier correctly classifies a movement as TEMPO to the number of times that the classifier classifies a movement as TEMPO. The recall is taken as the ratio of the number of times that the classifier correctly classifies a movement as TEMPO to the number of times that the user actually performs TEMPO.

In the aforementioned experiments, all subjects easily and correctly conducted TEMPO without prior training. Furthermore, during these experiments, no subject was observed to experience tongue fatigue or to accidentally bite his or her tongue.

## 5. Discussion

Figure 5 shows that the normalized measured value decreases when the subject performs TEMPO and increases when the tongue is returned. In other words, during TEMPO the measured waveform becomes U-shaped. Figure 5 also shows that the response to TEMPO is highly reproducible. Based on the ground truth shown in Figure 6, the measured waveforms corresponding to TEMPOs from all subjects have the same U shape. Although the data are not shown here, the data obtained during TEMPO were also highly reproducible in the other test subjects (B–E). The difference between subjects in the ground truth in Figure 6 depends on the length of time during which the tongue was kept pressed against the roof of the mouth. However, upon returning the tongue, the measured values were consistent with the exception of those obtained from subject E, which were about 20% lower than those measured before the tongue was moved. 

Based on the data shown in Table 1, the accuracy was 1.00 while resting and masticating and 0.99 while walking for all subjects. The precision was 1.00 for all subjects. Furthermore, the recall was 1.00 while resting and masticating in subjects A, B, and D, 0.90 while resting and 0.80 while masticating in subject C, and 0.70 while resting and masticating in subject E. While walking, the recall was 0.70, 0.40, 0.30, 0.30, and 0.70 for subjects A, B, C, D, and E, respectively, with an average of 0.48 and a standard deviation of 0.18.

In the experiments conducted here, PAP operation was performed successfully by all subjects tested while resting while and masticating. In contrast, PAP by performing TEMPO was found to be challenging while walking. As shown in Figure 7, walking significantly affects the measurements. This is likely due to the rocking motion of the data-transfer cable attached to the earphone-type sensor; in addition, the vibrations transmitted from the feet to the ear are superimposed onto the measurements obtained by the earphone-type sensor. Due to this interference, the correlation coefficients for the values obtained while walking were low and, therefore, the recall was low.

Together, these results show that TEMPO operation is highly reproducible while resting. Furthermore, while resting, masticating, and walking, the PAP was not erroneously signaled to start or stop the music if TEMPO is not performed by the user; the user does not erroneously bite the tongue even while walking; and the TEMPO can be performed without training. These findings indicate that TEMPO is suitable for the operation of a PAP. The earable TEMPO is compatible with equipment to handle voice information, because it can be integrated with an earphone. In addition, it is safe for everyday use, because only one ear is blocked, while the other can be used to remain aware of the surroundings. For this reason, the proposed approach can also be applied to the hands-free operation of one-ear Bluetooth headsets and hearing aids, which are conventionally operated by hand. Furthermore, the concept of the earable TEMPO will lead to the development of new ways of operating smartphones and wearable devices. Additionally, this can also be applied to devices that support people with disabilities of the hands or feet, or for workers that have both hands obstructed with tools and the like. Many applications can be considered in this way. When carrying out this study, we chose, from a large number of applications, those that can be applied to PAP operation devices. We did because the earable TEMPO can be integrated with earphones, and is compatible with PAPs, which are commonly used throughout the world.

As we stated with the concept in Section 2.1, we assume that PAP users are “doing something while” listening to music. When creating practical applications for the earable TEMPO, we would like to carry out a survey to discover whether this assumption is correct, as well as survey of what needs people have for the earable TEMPO.

Our future goal is to improve recall while walking by improving materials and algorithms and implementing a noise-reduction technology.

## 6. Conclusions

In this study, we developed the earable TEMPO that can be used for the hands-free operation of PAPs (starting and stopping music) by a simple motion of pressing the tongue against the roof of the mouth for about one second, which is referred to as TEMPO. In this approach, an earphone-type sensor placed in the right ear is used to optically measure changes in the shape of the external acoustic meatus when the tongue is pressed against the roof of the mouth. Based on this measurement result, the earable TEMPO identifies when the user performs TEMPO and accordingly operates the PAP by transmitting a one-bit control signal to either start or stop the music.

Here, the proposed system was tested on five subjects (men and women aged 22–58) while resting, masticating, and walking. The results showed that all subjects could reliably perform TEMPO. Shape variation of the external acoustic meatus that occurs during a TEMPO is highly reproducible and therefore is suitable for the operation of a PAP. Furthermore, the average accuracy was 1.00 while resting and masticating and 0.99 for walking; the precision was 1.00 in all experiments; and the average recall for the five subjects was 0.92 while resting, 0.90 while masticating, and significantly lower in comparison (0.48) while walking. The poor recall while walking is considered to be due to the rocking motion of the data-transfer cable that extends from the earphone-type sensor and the vibrations transmitted from the feet to the ears that influence the measurements of the earphone-type sensor. In future studies, to improve the recall while walking, we aim to develop a technique that removes noise due to the motion of the data-transfer cable and vibrations transmitted from the feet to the ears.

## Figures and Tables

**Table 1:** Results of the PAP operation experiment: Subjects A–E were tested while resting for 3 min, masticating 100 times, and walking for 80 s. A TEMPO was performed ten times in synchronization with the lighting of a timing-teaching LED as described in Section 3.3. The accuracy, precision, and recall were calculated from the obtained data.

Subject	Item	Accuracy	Precision	Recall
A	Rest	1.00	1.00	1.00
	Chewing	1.00	1.00	1.00
	Walk	0.99	1.00	0.70
B	Rest	1.00	1.00	1.00
	Chewing	1.00	1.00	1.00
	Walk	0.99	1.00	0.40
C	Rest	1.00	1.00	0.90
	Chewing	1.00	1.00	0.80
	Walk	0.99	1.00	0.30
D	Rest	1.00	1.00	1.00
	Chewing	1.00	1.00	1.00
	Walk	0.99	1.00	0.30
E	Rest	1.00	1.00	0.70
	Chewing	1.00	1.00	0.70
	Walk	0.99	1.00	0.70
Average	Rest	1.00	1.00	0.92
	Chewing	1.00	1.00	0.90
	Walk	0.99	1.00	0.48
Standard Deviation	Rest	0.00	0.00	0.12
	Chewing	0.00	0.00	0.13
	Walk	0.00	0.00	0.18
